# Advances in translational bioinformatics and population genomics in the Asia-Pacific

**DOI:** 10.1186/1471-2164-13-S7-S1

**Published:** 2012-12-13

**Authors:** Shoba Ranagathan, Sissades Tongsima, Jonathan Chan, Tin Wee Tan, Christian Schönbach

**Affiliations:** 1 Department of Chemistry and Biomolecular Sciences and ARC Centre of Excellence, Macquarie University, Sydney, NSW 2109, Australia; 2 Department of Biochemistry, Yong Loo Lin School of Medicine, National University of Singapore, Singapore 117597, Republic of Singapore; 3 National Center for Genetic Engineering and Biotechnology (BIOTEC), National Science and Technology Development Agency (NSTDA), Thailand Science Park, Pathumthani 12120, Thailand; 4 School of Information Technology, King Mongkut's University of Technology Thonburi, Bangkok 10140, Thailand; 5 Computational Resource Centre (A*CRC), A*STAR, Singapore 138632, Republic of Singapore; 6 Department of Bioscience and Bioinformatics, Kyushu Institute of Technology, Fukuoka 820-8502, Japan; 7 Biomedical Informatics Research and Development Center, Kyushu Institute of Technology, Fukuoka 820-8502, Japan

## Abstract

The theme of the 2012 International Conference on Bioinformatics (InCoB) in Bangkok, Thailand was "From Biological Data to Knowledge to Technological Breakthroughs." Besides providing a forum for life scientists and bioinformatics researchers in the Asia-Pacific region to meet and interact, the conference also hosted thematic sessions on the Pan-Asian Pacific Genome Initiative and immunoinformatics. Over the seven years of conference papers published in BMC Bioinformatics and four years in BMC Genomics, we note that there is increasing interest in the applications of -omics technologies to the understanding of diseases, as a forerunner to personalized genomic medicine.

## Background

Since 1998, the Asia-Pacific Bioinformatics Network (APBioNet) [1] has worked towards fostering bioinformatics as a scientific discipline in the Asia-Pacific region. The International Conference of Bioinformatics (InCoB) series of conferences has served as the annual APBioNet conference since 2002, spanning an audience form students to experts from the life, computing, -omics and biomedical sciences. The progress of bioinformatics in the Asia-Pacific region as documented in the editorials of previous InCoB supplements in *BMC Bioinformatics *and *BMC Genomics *Supplements have been summarized by Schönbach *et al.*[2], along with a detailed description of the 2012 conference at Bangkok, Thailand, Oct 3-5, 2012 and the review process for accepted conference papers, published in this supplement as well as in *BMC Bioinformatics *[2]. With the global trend towards personalized medicine, InCoB2012 supported presentations from the Pan Asia Population Genomics Initiative (PAPGI) [3]. A detailed report on the two special PAPGI sessions is presented here, while the immunoinformatics session has been reported in *BMC Bioinformatics *[2].

APBioNet's 11^th ^International Conference on Bioinformatics [4] was held in Bangkok, Thailand on Oct 3-5, 2012. with two satellite meetings the 3rd International Conference on Computational Systems Biology and Bioinformatics (CSBio2012) and the 3rd Winter Conference of the International Neural Networks Society (INNS-WC2012) co-hosted by Thailand's National Center for Genetic Engineering and Biotechnology (BIOTEC), as well as the King Mongkut's University of Technology Thonburi (KMUTT). Keynote addresses covered the development of bioinformatics as a research discipline in Thailand, comparative genomics of micobes and their global catalogue, computational drug design, genome-wide association studies, disease-based genome variations and the milestones and future of genomic medicine.

### Pan Asia Population Genomics Initiative (PAPGI) session report

InCoB2012 hosted a special forum, organized by PAPGI researchers. PAPGI, formerly known as the Pan Asian SNP Initiative (PASNP), is a collaborative project that strives to explain the Pan Asian continuous spectrum of phenotypic traits by deciphering the underlying population genomic diversity. In 2009, the consortium published the fundamental prediction of Asian migration history [5] using the genotyping information of 1,928 individuals. In this project, PAPGI researchers plan to use Next Generation Sequencing (NGS) to uncover other uncommon variants that could further unravel patterns in genomic diversity. Several teleconferencing meeting were conducted over the past year to iron out agreements and research protocols in order to smoothly execute the sequencing project as well as post analyses of the sequencing data. During the two special sessions at InCoB2012, nine key researchers from PAPGI delivered talks about research progress, focusing on a proposed computational workflow to analyze the data. In particular, several new bioinformatics analytical pipelines based on graph theory were proposed to analyze the enormous NGS data from large number of individuals. Based on the fact that self-reported origins may not be accurate, unsupervised clustering using genetic diversity information should be used to re-cluster data into genetically similar groups [6]. There are also discussions on the progress of the NGS data generated from Singapore, Malaysia, and Kuwait. Furthermore, the data from PASNP were reanalyzed in different context that explain the local adaptation of Pan Asian population using their admixture history [7].

During the discussions, several issues including ratification of consent agreement, sample selection criteria, collaboration with other countries/societies and external funding were raised. In particular, PAPGI will be officially affiliated to the Human Genome Organization (HUGO). The affiliation to the Asia Pacific Society of Human Genetics is being finalized. Since the samples used in PAPGI may be different from the ones used in the previous PASNP study and there are more countries, mainly from the Middle East participating in the project, issues about sample selection criteria was discussed and a general guideline for PAPGI sample selection will be drafted.

### Overview of InCoB2012 accepted papers

Of the 53 accepted proceedings papers 25 articles were published in the *BMC Bioinformatics *Vol. 13 Supplement 17 [2] while the remaining 28 articles in this issue are presented thematically.

#### Genomics

Piriyapongsa *et al. *[8] present iLOCi, a new approach for detecting epistasis in genome-wide association studies, while Jensen *et al. *[9] have predicted bacterial growth from genomic sequences, using a Bayesian approach. Chen *et al. *[10] have carried out bacterial whole genome sequencing to identify pathogenicity-related genesm while Chu *et al. *[11] have developed MitoCounter to quantitaively report mitochondrial DNA copy numbers, important for aging dosorders. snpTree [12] is a new rapid standardised and automatic SNP analysis approach for epidemiological studies while 454 pyrosequencing, coupled with bioinformatics analysis, has been effectively used to sequence the dengue virus quasispecies [13].

#### Transcriptomics

Garg and Ranganathan [14] have developed a helminth secretome database from EST sequences, whereas Chen *et al.*[15] present FastAnnotator for efficient transcript annotation. Menon *et al. *[16] have reported the large-scale annotation of the transcriptome of an economically important parasitic nematode. Vandenbon *et al. *[17] have systematically identified transcription factor pairs significantly co-occurring in a set of promoter sequences. Wu *et al. *[18] have uncovered novel multi-cancer differentially expressed gene candidates using meta-analytical biomarker search of EST expression data.

#### miRNA and sRNA

Khoo *et al. *[19] report the computational indentification and experimental validation of small RNAs (sRNAs) from *Burkholderia pseudomallei*, a soil bacterium which causes the disease melioidosis. Yang *et al. *[20] have proposed genetic buffering roles of for human microRNAs (miRNAs) on genome expression fluctuation. miRNAs can be used to design virus vaccines [21] while miRNAs and their targets can be rapidly identified even in plants [22]. Lee *et al. *[23] have developed a comprehensive analytical approach for the functional annotation of miRNAs, while Chang *et al. *[24] have analyzed miRNAs in breast cancer.

#### Proteins, ligands, docking and virtual screening

Ali *et al. *[25] have developed organism-specific substitution matrices for improved protein annotation, while Grover *et al. *[26] report mechanistic insights into the anti-leishmanial activity of prospective herbal drugs. Chiu *et al. *[27] report pharmaceutical motifs for rapid identification of target proteins.

#### Mutations

Kumar *et al. *[28] have engineered aggregation-prone recombinant proteins for substrate recognition by GroEL, while Wozniak *et al. *[29] have presented their results on finding drug resistance associated mutations in bacterial strains.

#### Pathways and networks

Praneenararat *et al. *[30] present NaviClusterCS for interactive and multi-scale network navigation in the big data era while Hsu and Yang [31] have uncovered pathway cross-talks based on functional relations between pathways. Hwang [32] has compared five methods of pathway-aggregation for gene expression data and report the best two. Li and Li [33] have identified disease genes by merging genotype and phenotype data using multigraph networks.

#### Emerging technologies and applications

To leverage on the newly developed cloud computing technology, Chang *et al. *[34] have developed a *de novo *next-generation sequence assembler. An application of modern genome informatics to marine ecosystems is reported by Somboonna *et al. *[35], in their study of metagenomic profiles of free-living prokaryotes and eukaryotes in the coastal areas of Sichang Island in Thailand.

The best paper title was awarded to Li and Li for their advancement of disease gene identification using a new random walk model that allows cross-walking between phenotype and gene networks [33]. The runners-up were Teo *et al. *[36] and Abbas *et al. *[37], in the *BMC Bioinformatics *supplement.

## Future Conferences

APBioNet participated recently in the 1st International Conference in Translational Biomedical Informatics (ICTBI; http://ictbi.imed-cn.org/) in Taicang, China. To follow the rising interest in biomedical and translational bioinformatics InCoB2013 will be hosted jointly with the 2nd ICTBI and is scheduled to be held September 18-21, 2013 in Suzhou, China. We also hope to support PAPGI session, bringing population genomics together with translational bioinformatics, in the quest for personalized genomics.

## Competing interests

The authors were organizers, co-chairs, and/or session chairs of InCoB2012. TWT is a founding Director of Asia Pacific Bioinformatics Network, Ltd. All authors declare they have no other conflict of interest.

## Authors' contributions

SR and CS wrote this editorial. SR, ST, TWT and CS served as co-editors for the InCoB2012 supplement issues with SR as the lead editor. CS and SR managed the manuscript submission, peer-review and editorial decision processes as superchairs of EasyChair Conference System.
